# The chloroplast genome of *Adiantum reniforme* var. *sinense*, an endangered fern endemic to China

**DOI:** 10.1080/23802359.2020.1815601

**Published:** 2020-09-01

**Authors:** Jingfang Chen, Yongmei Chen, Renchao Zhou, Ying Liu

**Affiliations:** a State Key Laboratory of Biocontrol and Guangdong Provincial Key Laboratory of Plant Resources, School of Life Sciences, Sun Yat-sen University, Guangzhou, China; b School of Chemical Engineering, Sichuan University of Science and Engineering, Zigong, China

**Keywords:** *Adiantum reniforme* var. *sinense*, chloroplast genome, phylogenetic analysis

## Abstract

*Adiantum reniforme* var. *sinense*, a rare and endangered fern endemic to Chongqing, China, has been listed as one of the National Key Protected Wild Plants (Class I) in China. Here we assembled its chloroplast genome (cp genome) using Illumina sequencing reads to offer a genetic resource for future conservation genetic studies. The complete cp genome of *A. reniforme* var. *sinense* was 150,109 bp in length with an overall GC content of 42.85%. It contains a large single copy (LSC) region of 83,274 bp and a small single copy (SSC) region of 21,459 bp, separated by a pair of inverted repeat regions (IRs) of 22,688 bp each. The cp genome contains 84 protein coding genes, 28 tRNA genes, four rRNA genes, and one pseudogene (*ψrps16*). Phylogenetic analysis showed that *A. reniforme* var. *sinense* clustered together with four other *Adiantum* species and was sister to *A. capillus-veneris*.

*Adiantum reniforme* var. *sinense* Y.X.Lin (Pteridaceae, Vittarioideae) is a rare and endangered fern species with a narrow distribution area in Chongqing, China (Wang et al. 2015). Because of its rarity, it has been listed as one of the National Key Protected Wild Plants (Class I) (http://www.iplant.cn/info/Adiantum reniforme var. sinense). So far, available genetic information for this species is very limited. Here, the cp genome of *A. reniforme* var. *sinense* was assembled and characterized to provide a useful genomic resource for its conservation genetics studies.

Fresh leaves of an *A. reniforme* var. *sinense* individual were sampled from Shizhu, Chongqing, China (108°10′0″E, 30°23′29″N) for genomic DNA isolation. The voucher specimen (Zhou20190803) was deposited in the herbarium of Sun Yat-sen University (SYS). A genomic library with an insert size of 350 bp was constructed and then sequenced on an Illumina HiSeq X 10 platform. Approximately 16.67 Gb of paired-end 150 bp short reads were generated and then used to assemble the chloroplast genome with GetOrganelle v1.6.4 (Jin et al. 2019) under default parameters. The assembled cp genome was annotated using GeSeq (Tillich et al. 2017) with four other *Adiantum* cp genomes (GenBank accessions: NC_040172, NC_040209, NC_004766, and NC_037478) as the references, followed by manual adjustments. The annotated cp genome sequence has been deposited into GenBank with the accession number MT733232.

The complete cp genome of *A. reniforme* var. *sinense* was 1,50,109 bp in length with an overall GC content of 42.85%, containing a large single copy (LSC) region of 83,274 bp, a small single copy (SSC) region of 21,459 bp, and a pair of inverted repeat region (IRs) of 22,688 bp each. The cp genome contains 84 protein-coding genes, 28 transfer RNA (tRNA) genes, four ribosomal RNA (rRNA) genes and one pseudogene (*ψrps16*).

11 species from the subfamily Vittarioideae were included in the phylogenetic analysis, with *Pecluma dulcis* (Polypodiaceae) as an outgroup. The concatenated sequences of 81 shared protein-coding genes were aligned using MAFFT (Katoh and Standley 2013). A maximum likelihood phylogenetic tree was reconstructed using RAxML (Stamatakis 2014), employing the GTR + G model with 1000 bootstrap iterations (-m GTRGAMMAX | -# 1000). As shown in Figure 1, A. *reniforme* var. *sinense* clusters with four other *Adiantum* species and is sister to *A. capillus-veneris* under 100% bootstrap support values. The cp genome of *A. reniforme* var. *sinense* offers a valuable genomic resource for future conservation genetics studies.

**Figure 1.**
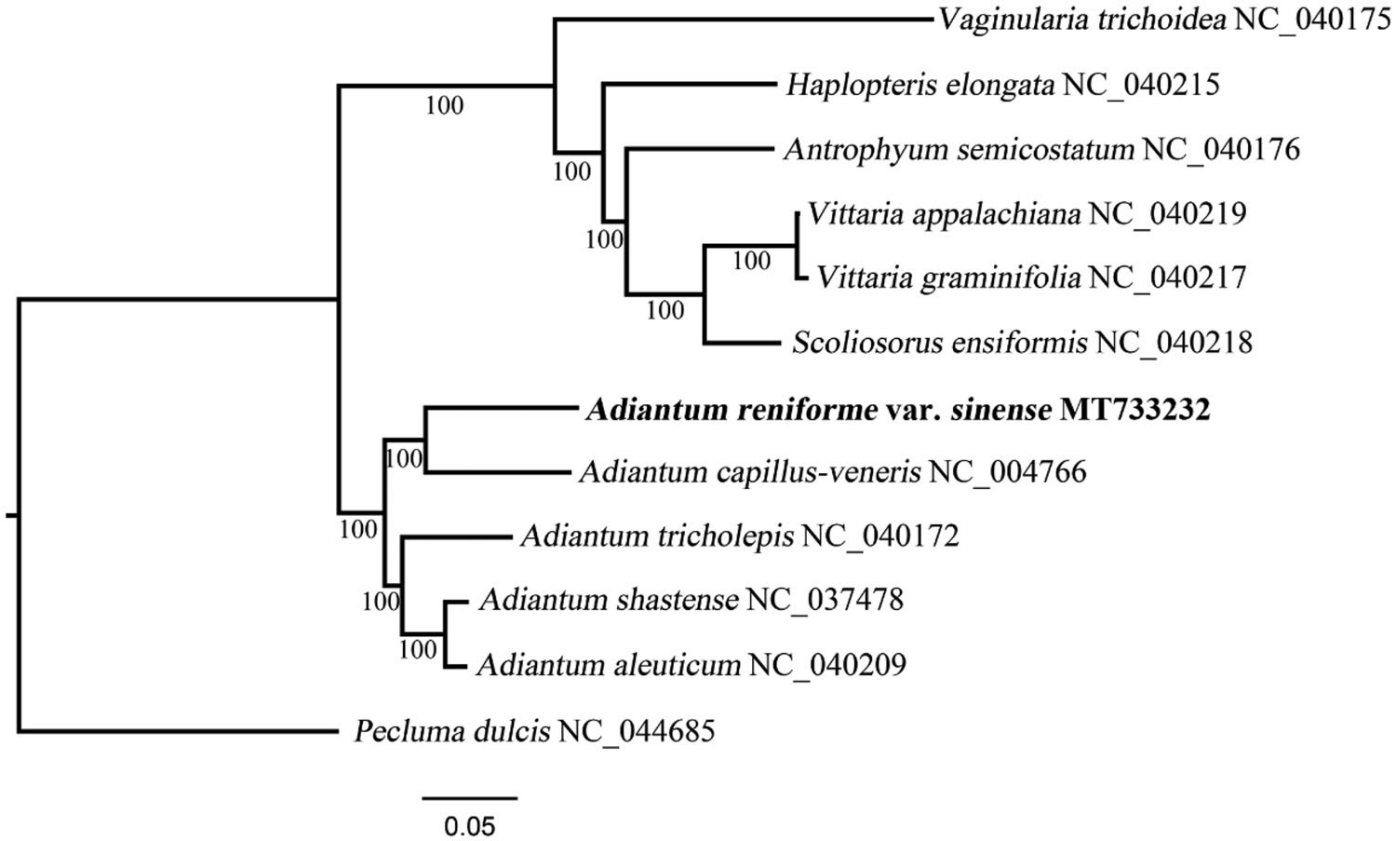
A maximum likelihood tree based on 81 protein-coding genes of 12 cp genomes to show the phylogenetic position of *Adiantum reniforme* var. *sinense*. Bootstrap values are indicated at each node.

## Data Availability

The chloroplast genome of the *A. reniforme* var. *sinense* was submitted to Genbank under accession number MT733232 (https://www.ncbi.nlm.nih.gov/).
